# Large lipoma of the spermatic cord presenting as post-operative recurrent hernia in a middle aged gentleman: a case report

**DOI:** 10.4076/1757-1626-2-8500

**Published:** 2009-08-25

**Authors:** Ravindran Vashu, Manisekar Subramaniam

**Affiliations:** Hospital Alor Star, Jalan Langgar, Alor StarKedah 05250Malaysia

## Abstract

A gentleman who presented with a left inguinal hernia was operated and treated by hernioplasty. After a few years he presented with a clinical scenario of recurrent hernia. During surgery the lump was found to be a large lipoma that was not documented and found during the first operation.

## Introduction

Lipoma of the cord is the most common benign tumour of the spermatic cord and a common condition of the male population [1]. It is frequently under-diagnosed and ignored because of its benign course. Clinical series have reported an incidence of 22.5% during inguinal hernia surgery [2]. The term ‘large lipoma’ has yet to be clearly defined. However, lipoma exceeding 10 cm in size has been described to be large in literatures [3]. We are reporting a case of a large spermatic cord mimicking a recurrent hernia.

## Case presentation

**Figure 1.**
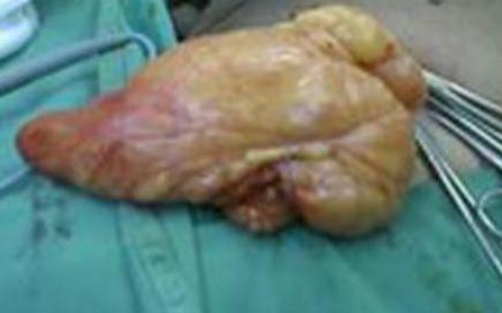
Giant lipoma delivered from the inguinal region.

**Figure 2.**
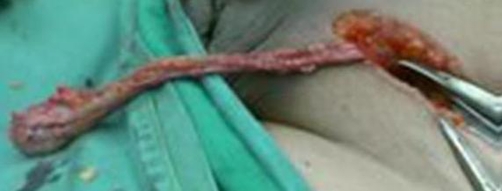
Long redundant cord visible after removal of the lipoma which was then fixed.

A 52-year-old Malay male presented 32 years ago with a left inguino-scrotal hernia. A left inguinal herniorraphy was performed uneventfully and there were no other abnormalities. 10 years ago, his swelling recurred. He was operated for recurrent hernia. During surgery, he had a recurrent inguinal hernia and herniorraphy was performed. Post-operatively, he was asymptomatic until 5 years ago when started developing painless recurrent inguinal mass of a similar nature. It was initially reducible and was ignored by patient. However about 1 month prior to surgery, the mass became irreducible. There was no associated pain or obstructive bowel symptoms. Attempts to reduce the swelling were not successful. On table, an incision was made over the old scar and explored. There was no hernial sac or recurrences. The spermatic cord was isolated and traced distally into the scrotum. A large lipoma was seen arising from the inguinal cord (Figure 1). It was dissected and excised. The cord was long and redundant (Figure 2). The cord structures were preserved and no signs of invasion or malignancies were seen. Orchidopexy was performed and abdomen closed in the usual manner. He was well upon discharge. He was followed up in the clinic and post-operatively uneventful after discharge. He was discharged to be followed up at the primary care and has been problem free for almost 2 years since surgery. Histopathology reports confirmed the diagnosis of lipoma.

## Discussion

Large lipomas of the cord remain a poorly defined entity. Its clinical importance, among others, is in the false diagnosis of an inguinal hernia [4]. Heller et al in his post mortem studies revealed that lipoma of the cord may be a common condition among the adult male population [5]. However the age studied, 24 to 92 years old, did not demonstrate its chronological growth pattern and its influence on the natural history of the disease. The etiology of this condition is largely unknown but a developmental origin has been proposed. Most lipomas are found incidentally during hernia surgery. The close relationship between cord lipomas and large inguinal hernias has been demonstrated [6]. The advent of laparoscopic surgery had demonstrated that the prevalence of this condition may be an underestimation [1]. Due to its indolent nature, it is frequently overlooked [7]. Excision of the tumour usually results in an uneventful recovery. Our report suggests that during the first hernia surgery, there were no documentation of a lipoma of the cord. Post operatively, a large size had grown into the left hemi- scrotum. The peculiarity of this report lies in its growth that has taken place in the post operative period to a considerable size and the pitfall to correct diagnosis. Though anecdotal, this may suggest that the origin may not be purely congenital but possibly multi-factorial. The factors that may influence its growth, such as age, previous surgery, pre/co-existing hernia and the extra peritoneal fat are beyond the interest of this report. We recommend further research to look more into this. The entity of ‘giant’ lipoma of the cord is also a descriptive term and may need to be well defined.

## Conclusion

Recurrent hernias are common but a giant lipoma may present in a similar manner and cause an incorrect diagnosis. The behaviour of this tumour needs to be investigated further.
